# Inhibition of ATG7 promotes orthodontic tooth movement by regulating the RANKL/OPG ratio under compression force

**DOI:** 10.1515/med-2025-1252

**Published:** 2025-09-12

**Authors:** Zhenjin Yang, Mingzhu Chen, Xianmin Liao, Liya Ma, Cun Liang, Yanjie Li, Jiangtian Hu

**Affiliations:** Department of Orthodontics, Kunming Medical University Affiliated Stomatological Hospital, Kunming, Yunnan, 650106, China; Yunnan Key Laboratory of Stomatology, Kunming, Yunnan, 650106, China; Stomatology Center, The First People’s Hospital of Yunnan Province, Kunming, Yunnan, 650500, China; Department of Orthodontics, Kunming Medical University Affiliated Stomatological Hospital, Building C, Hecheng International, No. 1088 Middle Haiyuan Road, Kunming, Yunnan, 650106, China

**Keywords:** ATG7, autophagy, orthodontic tooth movement, hPDLSCs, RANKL/OPG

## Abstract

**Objective:**

Autophagy serves as a protective mechanism in response to mechanical stress during OTM. However, the role of ATG7, a key regulatory gene in autophagy, in modulating periodontium remodeling during OTM remains unclear. This study aims to investigate the potential modulation of periodontium remodeling by ATG7 under compression force.

**Materials and Methods:**

HPDLSCs and a rat OTM model were used as *in vitro* and *in vivo* systems to study the effect of compressive stress on autophagy and osteoclast-related markers. To investigate the role of ATG7, hPDLSCs with ATG7 knockdown and ATG7^+/−^ rats were used in this study.

**Results:**

Compression force activates autophagy and increases the RANKL/OPG ratio. ATG7 knockdown significantly suppresses autophagy in hPDLSCs, while the RANKL/OPG ratio is markedly elevated. Under compression stress, hPDLSCs-siATG7 markedly enhanced RANKL/OPG expression. In the rat OTM model, autophagy was significantly activated in the periodontium on the compression side. Compared to wild-type SD rats, ATG7^+/−^ rats exhibit reduced autophagy-related protein expression, an increased RANKL/OPG ratio, and accelerated tooth movement.

**Conclusions:**

Under compression force, inhibition of ATG7 expression significantly increases the RANKL/OPG ratio both *in vivo* and *in vitro*, which is accompanied by an increased rate of orthodontic tooth movement.

## 1 Introduction

Orthodontic treatment is gaining popularity due to the increasing public demand for oral care [1]. However, the extended duration of orthodontic treatment remains a major obstacle, discouraging many patients from seeking interventions. Clinically, prolonged orthodontic therapy may lead to iatrogenic complications, including root resorption, gingival recession, or demineralization of enamel [2,3]. Consequently, expediting the movement of teeth during orthodontic therapy emerges as a shared objective for both practitioners and patients. To effectively control tooth movement, it is essential to understand the underlying mechanisms of orthodontic tooth movement (OTM). OTM involves the application of mechanical forces to periodontal tissues, which are then converted into biological signals within cells. This process induces tissue remodeling through cell activation and differentiation, with bone resorption occurring on the compressed side and bone formation on the tension side, ultimately facilitating the movement of teeth [4]. During this cascade, mechanical signals are converted into intracellular biological signals, triggering responses such as inflammation [5], hypoxia [6], and autophagy [7]. Among these responses, autophagy plays a crucial role in regulating OTM, marking it one of the prominent research areas in recent years.

Autophagy encompasses cellular degradation and recycling processes, which maintain cell homeostasis by removing damaged and dysfunctional organelles. In mammals, autophagy can be divided into three types based on the different ways substrates are transported to lysosomes: macroautophagy, chaperone-mediated autophagy (CMA), and microautophagy [8]. In this study, the term “autophagy” primarily refers to macroautophagy. Autophagy, as an important regulatory factor in tooth movement, plays a significant role in the process of OTM. Numerous studies related to autophagy and OTM have been reported [9,10,11,12,13], suggesting that physiological tooth movement can induce increased autophagic activity on the pressure side of the tooth. This process is associated with a significant upregulation of inflammatory cytokines such as IL-1, IL-6, and TNF-α, as well as an increase in the ratio of receptor activator of nuclear factor kappa-B ligand (RANKL) to osteoprotegerin (OPG), which in turn promotes bone resorption. These events contribute to alveolar bone remodeling, ultimately facilitating tooth movement. During this process, both *in vivo* and *in vitro* studies have demonstrated that the application of autophagy inhibitors significantly enhances the expression of inflammatory factors, promotes the recruitment of osteoclasts, reduces bone density, and accelerates tooth movement. Conversely, excessive autophagy activation after using autophagy activators may suppress tooth movement by dampening the inflammatory cascade reaction and interfering with osteoclast recruitment, ultimately resulting in a slowing down of the tooth movement speed.

Autophagy related 7 (ATG7) serves as an E1 activating enzyme for conjugation, ultimately forming the ATG12–ATG5–ATG16L1 complex, which participates in membrane elongation of the phagophore. At the same time, ATG7 also promotes lipidation of protein LC3-I to generate LC3-II. LC3-II is present on the inner and outer membranes of autophagosomes, and directly or through selective autophagy receptor proteins sequesters cytoplasmic targets into autophagosomes [14,15]. It is evident that ATG7 is an indispensable component of the autophagy process, and autophagy is closely associated with bone homeostasis. Conditional knockout of ATG7 in mice (autophagy-deficient mice) has been shown to impair the functions of osteoblasts and osteoclasts, disturb bone metabolic homeostasis, and result in a pronounced phenotype characterized by reduced bone mass [16,17,18]. Moreover, ATG7 can influence the differentiation of BMSCs toward bone formation [19]. However, it remains unclear whether ATG7 influences the differentiation potential of human periodontal ligament stem cells (hPDLSCs), which are key seed cells involved in alveolar bone remodeling under mechanical stress. Furthermore, its potential role in regulating OTM *in vivo* has not been fully elucidated.

In summary, in this study, we plan to investigate the role of ATG7 in regulating autophagy, and its downstream effects on osteoclastogenesis and OTM speed, providing scientific evidence for the mechanism regulating tooth movement. This study may offer new insights into optimizing orthodontic treatment by targeting autophagy-related pathways involved in periodontal remodeling.

## 2 Materials and methods

### 2.1 Isolation and characterization of hPDLSCs

The experiments were approved by the Medical Ethics Committee of the Affiliated Stomatology Hospital of Kunming Medical University (Approval number: KYKQ2023MEC026), and all involved patients and their guardians provided consent. The volunteers in the project were patients aged 12–18 years whose healthy premolars were to be extracted for orthodontic treatment. The harvested teeth were separated and rinsed with phosphate-buffered saline (PBS, Beyotime, Shanghai, China) three times, and periodontal ligament tissues were scraped from the middle 1/3 of the root with a scalpel. They were then incubated in Dulbecco's modified Eagle’s medium/Nutrient Mixture F-12 (DMEM/F12; Biological Industries, Beit Haemek, Israel) medium containing 20% fetal bovine serum (FBS, Gibco, Carlsbad, CA, USA) at 37°C under 5% CO_2_. The media was changed every 3 days, and the cells of passage 3–5 were used for the experiment.

After three passages, the cells were harvested for identification. For osteogenic and adipogenic differentiation, the cells were cultured in 6-well plates (1 × 10^5^ cells/well). After reaching 80% confluency, the cells were oriented and induced using osteogenic differentiation medium (Cyagen, Suzhou, China) and adipogenic differentiation medium (Cyagen, Suzhou, China) for 2 or 3 weeks, respectively. After differentiation, cells were stained with Alizarin red or Oil Red O and observed under an inverted microscope (Olympus, Tokyo, Japan). Immunofluorescence staining was used to detect cell surface markers. Briefly, cells were inoculated at 1 × 10^5^ cells/well on a 6-well. At 80% confluence, cells were fixed with 4% paraformaldehyde, washed with PBS, and stained with the following antibodies: anti-Vimentin (Zen-Bio, Chengdu, China) and anti-Nestin (Zen-Bio, Chengdu, China), according to the manufacturer's protocols, and then visualized with Fluorescein-Conjugated Goat anti-Rabbit IgG (ZSGB-Bio, Beijing, China). 6-Diamidino-2-phenylindole (DAPI, Beyotime, Shanghai, China) solution was used for the staining of nuclei. Images were obtained using a laser scanning confocal microscope (Nikon, Tokyo, Japan). For flow cytometric analysis, the cells were resuspended in cold PBS containing 2% FBS at a concentration of 1 × 10^6^ cells/mL prior to adding the following monoclonal antibodies: CD29-PE, CD44-PE, CD90-PE, CD105-PE, CD34-FITC, and CD45-FITC (BD Biosciences, San Jose, CA, USA). The unmarked cells were used as a negative control. Finally, the stained cells were analysed using BD Accuri^®^ C6 (BD Biosciences, San Jose, CA, USA) and FloMax^®^ software (Beckman Coulter, Brea, CA, USA). Cell clone formation ability was tested using the plate clone formation assay.

### 2.2 Application of compressive stress

HPDLSCs were seeded into 6-well plates at 10^5^ cells/well and cultured to confluence. The cells were continuously compressed using a uniform compression method similar to that previously described [20]. Briefly, a circular industrial glass plate with a diameter of 33 mm and a weight of approximately 17 g was placed over the cell layer to apply a constant mechanical pressure. When the glass was placed vertically in the 6-well plate, it generated a compressive stress of about 2 g/cm^2^. Cells were immediately collected after applied stress for 0, 6, 12, 18, and 24 h.

### 2.3 Cell transfection

HPDLSCs were seeded into 6-well plates at 10^5^ cells/well and cultured in medium without antibiotics. Upon reaching 60–70% confluence, a final concentration of 50 nM siRNA (Zixi Biotechnology, Beijing, China) was transfected into each group of cells. The siRNA and transfection reagent Lipofectamine™ 3000 (Thermo Fisher Scientific, Waltham, MA, USA) were diluted with Opti-MEM (Gibco, Carlsbad, CA, USA) separately, then combined them gently, and allowed to incubate for 20 min at room temperature. The resulting transfection complex was added to the 6-well plate and cultured at 37°C under 5% CO_2_ for 5 h. Then the transfection mixture was replaced with fresh culture medium, and the cells were further incubated for at least 24 h before subsequent experiments. The primer sequences corresponding to siRNA1 targeting ATG7 are F: GACAUUAAGGGUUAUUACU (dT)(dT), R: AGUAAUAACCCUUAAUGUC (dT)(dT).

### 2.4 Transmission electron microscopy (TEM)

The TEM was performed on hPDLSCs cultured on coverslips to observe autophagosome generation. The samples were fixed overnight at 4°C using 2.5% glutaraldehyde in 0.1M PBS, pH 7.2 (Powerful Biology, Wuhan, China), then washed with 0.1M PBS three times. Afterwards, samples were postfixed with 1% OsO4 for 2 h at 4°C, then washed with ddH_2_O three times, followed by 30, 50, 70, 90, and 100% ethanol dehydration and acetone transition for 5 min, then embedded in SPI pon 812 resin, and polymerization at 60°C for 48 h. After polymerization, 60 nm ultrathin sections were made using a Leica EM UC7 ultramicrotome. Ultrathin sections were then loaded onto Cu grids and double-stained with 2% uranyl acetate (Merck KGaA, Darmstadt, Germany) and lead citrate (Sigma-Aldrich, St.Louis, MO, USA) before observations employing a JEM-1400 Plus transmission electron microscope (JEOL Ltd, Tokyo, Japan) at 80 kV.

### 2.5 Western blotting

Western blotting was performed as previously described [21]. Briefly, hPDLSCs were lysed with radioimmuno-precipitation assay lysis buffer (Solarbio, Beijing, China). The protein levels were quantified using a Bradford assay kit (Beyotime, Shanghai, China). The antibodies used were the following: ATG7 (10088-2-AP, Proteintech, Wuhan, China), Beclin-1 (11306-1-AP, Proteintech, Wuhan, China), LC3 (14600-1-AP, Proteintech, Wuhan, China), Anti-SQSTM1/p62 (GB11531, Servicebio, Wuhan, China), RANKL (23408-1-AP, Proteintech, Wuhan, China), OPG (GXP366016, GenXspan, Alabama, USA), and GAPDH (60004-1-lg, Proteintech, Wuhan, China).

### 2.6 RT-qPCR

According to the manufacturer's protocol, total RNA was isolated using the TaKaRa MiniBEST Universal RNA Extraction Kit (Takara, Osaka, Japan). Total RNA was reverse-transcribed into cDNA using the PrimeScript™ RT Master Mix (Perfect Real Time) (Takara, Osaka, Japan), and qRT-PCR was conducted using a QuantStudio™ Real-Time PCR System (Thermo Fisher Scientific, Waltham, MA, USA) with the Taq Pro Universal SYBR qPCR Master Mix (Takara, Osaka, Japan). Relative expression levels were calculated using the 2^−ΔΔCt^ method. The primers were synthesized by Sangon Biotech (China, Table 1).

**Table 1:** Primers for RT-qPCR

Sequence	Name	Length (bp)
CGGTGATAATAGAACGATACAA	*LC3 (H)-F*	75
GGTCAGGTACAAGGAACT	*LC3 (H)-R*	75
TACAGAGTATCTTCAACTAATG	*RANKL (H)-F*	164
CTCCAGACCGTAACTTAA	*RANKL (H)-R*	164
AATGTGGAATAGATGTTACC	*OPG (H)-F*	94
TCTACCAAGACACTAAGC	*OPG (H)-R*	94
CAGATGGAGTCGGATAAC	*p62 (H)-F*	90
CTGGAGTTCACCTGTAGA	*p62 (H)-R*	90
GCTCTTCCTTACTTCTTA	*ATG7 (H)-F*	104
ATTGTTATCTTCGTCCTT	*ATG7 (H)-R*	104
GTGGAATGGAATGAGATTA	*beclin-1 (H)-F*	107
TAAGGAACAAGTCGGTAT	*beclin-1 (H)-R*	107
TTGCCCTCAACGACCACTTT	*GAPDH (H)-F*	120
TGGTCCAGGGGTCTTACTCC	*GAPDH (H)-R*	120

### 2.7 Establishment of the OTM model in SD rats

The animal experiment was approved by the Medical Ethics Committee of Kunming Medical University (Approval number: KMMU20231513), and the animal studies adhered to the international guidelines of ARRIVE. A total of 48 SD rats (male, 6–7 weeks old) divided into 6 groups were used in this study. A tooth movement model was established on the left maxillary first molar. The molar tooth movement was established as previously described [22]. All rats were anesthetized by intraperitoneal injection of 3% pentobarbital sodium, and a 50 g orthodontic force was applied using a nickel–titanium coil spring (Smart Advanced Material Tech Co., Ltd., Jiangsu, China), with the force measured by a force gauge (Xihu Biom, Hangzhou, China) prior to securing the spring with light-cured composite resin (3M, St. Paul, MN, USA). During the experiment, the behavioral changes of the rats were observed and recorded daily. Then, the rats were euthanized by over injection of pentobarbital sodium on days 0, 1, 3, 5, 7, and 14, and the specimens were collected.

### 2.8 Identification of the ATG7^+/−^ rats

The genetically modified rats used in the experiment were established by Syagen Biotechnology Company (Syagen Technology, Inc., Tustin, CA, USA) using CRISPR/Cas-mediated genetic engineering technology to generate the ATG7^+/−rat^ model. The gRNA and Cas9 mRNA targeting the ATG7 gene of SD rats were co-injected into zygotes, and the zygotes were then transferred to pseudopregnant rats. Positive F0 heterozygous rats were identified by PCR and sequencing. F0 heterozygous rats were bred with wild-type rats to obtain identified positive F1 heterozygous rats. F1 rats from the same F0 rat with consistent genotypes were selected and bred to obtain F2 rats. Offspring were genotyped by PCR using primer sequences, and the primer sequences are listed in Table 2. In wild-type ATG7^+/+^ rats, only the 592-bp PCR product can be detected, and in homozygous ATG7^−/−^ rats, only the 840-bp PCR product can be detected. Both 592-bp and 840-bp PCR products were detected in heterozygous ATG7^+/−^ rats. The heterozygous ATG7^+/−^ rats were selected for the experiment. A total of six heterozygous ATG7^+/−^ SD rats and six wild-type ATG7^+/+^ SD rats were used in this study.

**Table 2:** Primer sequences for the identification of genotype

Name	Sequence
Rat Atg7-F	ACACTGATATACGGTTAGCCGTTTAGAGAAA
Rat Atg7-R	GGTTGGTGGAGAGACCTATAAGGATGC
Rat Atg7-He/Wt-F	ACCATCGATTTGAAACTTAAACTTC

### 2.9 Micro-CT analysis

The collected samples were fixed using 4% paraformaldehyde for 24 h. Next, a NEMO^®^ NMC-100 micro-CT system (PINGSENG Healthcare Inc., Kunshan, China) was used to scan the maxillary molar regions with the following parameters: 90 kV source voltage and 60 μA source current. The data were reconstructed, and the tooth movement distance was measured from the distal surface of the first molar to the mesial surface of the second molar in each specimen using the Avatar 1.5.0 software (PINGSENG Healthcare Inc., Kunshan, China).

### 2.10 Histological analysis

The samples were fixed using 4% paraformaldehyde for 24 h, decalcified for 4 weeks, dehydrated using a graded series of ethanol, and embedded in paraffin wax. Embedded sections were prepared for hematoxylin and eosin (H&E) staining, tartrate-resistant acid phosphatase (TRAP) staining, and immunohistochemistry staining. The TRAP staining was performed according to the test kit instructions (tartrate-resistant acid phosphatase Assay, Beyotime, China). The immunohistochemical staining was performed according to a previously published method [23]. Sections were incubated with the following antibodies: ATG7 (10088-2-AP; Proteintech, Wuhan, China), Beclin-1 (11306-1-AP; Proteintech, Wuhan, China), LC3 (14600-1-AP; Proteintech, Wuhan, China), Anti-SQSTM1/p62 (GB11531; Servicebio, Wuhan, China), RANKL (23408-1-AP; Proteintech, Wuhan, China), and OPG (GXP366016; GenXspan, Alabama, USA). Immunohistochemical-stained sections, positive staining sites, were analyzed by the Image-Pro Plus version 6.0 software. Although eight or six animals per group were initially enrolled, only three samples per group were ultimately included in the statistical analysis. The remaining samples were excluded due to predefined technical criteria such as tissue damage, poor section quality, or loss during processing. Importantly, data exclusion was not based on experimental outcomes, and only samples meeting objective quality standards were analyzed.

### 2.11 Statistical analysis

All experimental data were statistically analyzed using SPSS 19.0 and GraphPad Prism 9.0.0. Independent sample *t*-test was used for comparison between two groups, one-way ANOVA was used for comparing three or more groups, and normality tests were performed before performing statistical analysis. Statistical significance was set at *P*＜0.05.

## 3 Results

### 3.1 Application of mechanical stress upregulated ATG7 and autophagy-related proteins on the compressive side of the OTM-rat model

**Figure 1:**
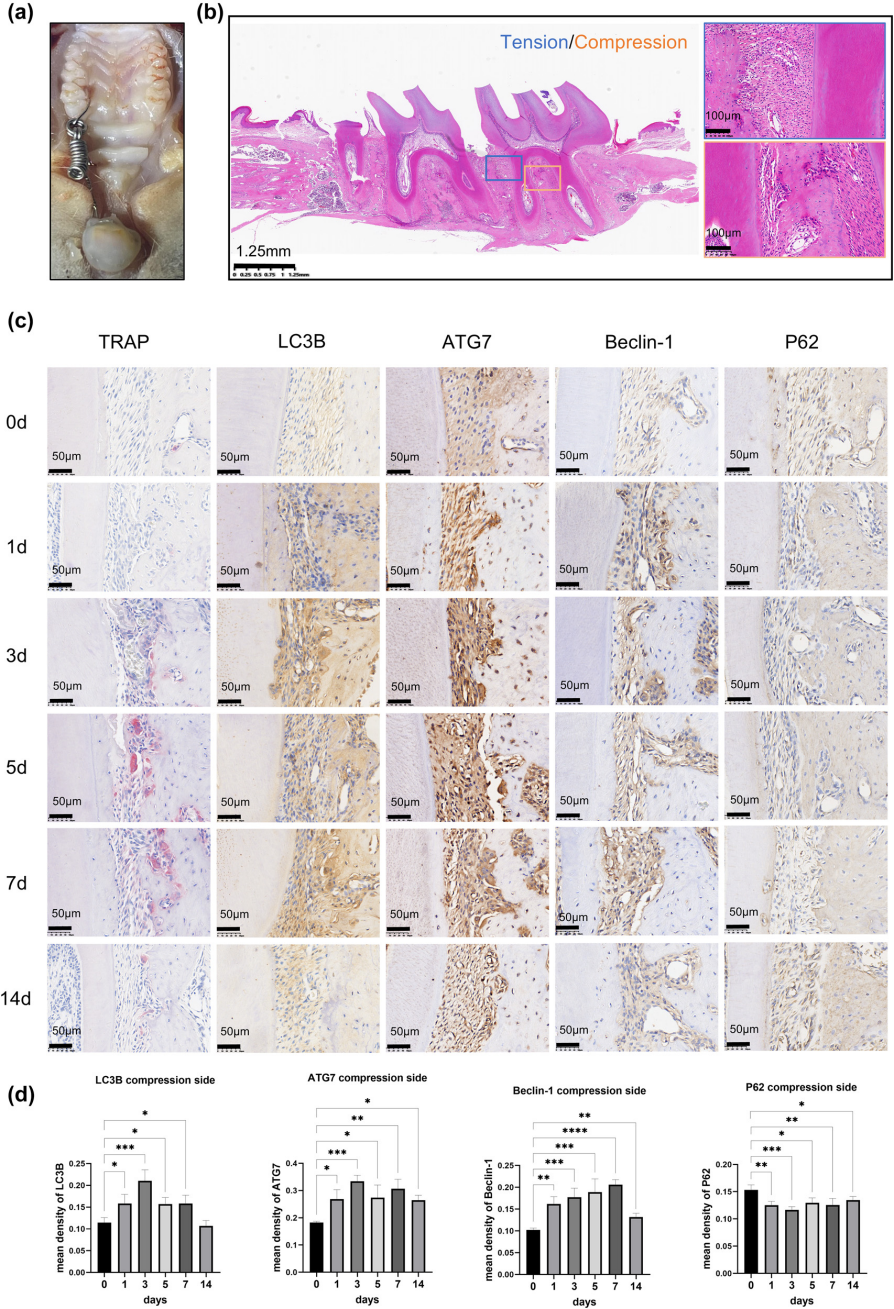
The application of mechanical stress upregulated autophagy-related proteins in the compression side of the OTM-rat model. (a) The model of tooth movement was established on the left maxillary first molar in rats. (b) H&E staining of OTM tissue section. The blue box represents the tension stress side, and the yellow box represents the compressive stress side of OTM. (c) and (d) The OTM model of SD rats was established for 0, 1, 3, 5, 7, and 14 days. The TRAP staining results showed that the red-stained multinucleated giant cells expressed the highest at days 5 and 7. Immunohistochemistry staining of autophagy-related proteins LC3B, ATG7, and Beclin-1 was significantly upregulated, while P62 was downregulated on the compression side of the periodontal ligament tissues. *n* = 3 per group; samples were selected based on the technical quality for histological analysis. *p < 0.05; **p < 0.01; ***p < 0.001; ****p < 0.0001.

A tooth movement model was established on the left maxillary first molar (Figure 1a), and the application of mechanical force induces both tension and compression zones around the tooth roots. Consequently, the periodontal ligament space was stretched on the tension side and constricted on the compression side (Figure 1b). TRAP staining demonstrated that red-stained multinucleated giant cells began to appear on the compressive side by the third day, peaking between days 5 and 7 (Figure 1c). Immunohistochemical results showed that under the application of orthodontic force, autophagy-related proteins LC3B, ATG7, and Beclin-1 were significantly upregulated on the pressure side of the periodontal ligament tissues, while P62 was downregulated. The expression of ATG7 fluctuated and attained its first peak at day 3 (Figure 1c and d).

### 3.2 Effect of compressive stress on the autophagy and RANKL/OPG expression in hPDLSCs

**Figure 2:**
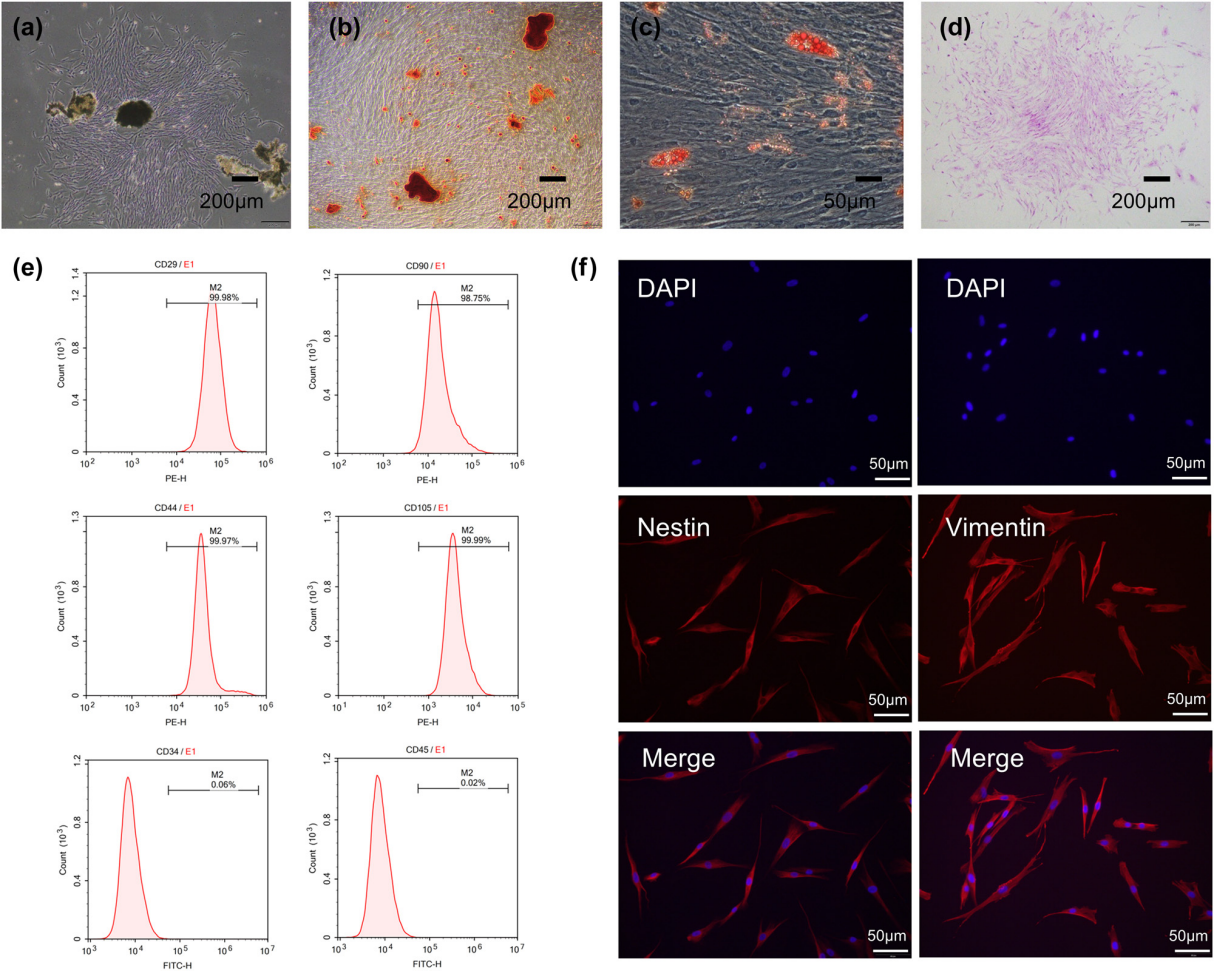
Culture and identification of hPDLSCs. (a) Primary cells cultured by periodontal ligament tissue. (b) Alizarin Red S staining of hPDLSCs after being cultured in osteogenic differentiating medium. (c) Oil Red O staining of hPDLSCs after being cultured in adipocytic differentiating medium. (d) Cell colony formation was observed after crystal violet staining. (e) Flow cytometric analysis of surface markers in hPDLSCs, CD29-PE (99.98%), CD90-PE (98.75%), CD105-PE (99.99%), CD44-PE (99.97%), CD34-FITC (0.06%), and CD45-FITC (0.02%). (f) Vimentin expression and Nestin expression in hPDLSCs.

We isolated and cultured primary cells from detached periodontal ligament tissue (Figure 2a), and osteogenic and adipogenic differentiation results showed that the cells possessed good osteogenic ability (Figure 2b) and adipogenic ability (Figure 2c). Through cloning formation experiments, we demonstrated that the obtained cells had good cloning formation ability (Figure 2d). Subsequently, we conducted flow cytometry analysis, which revealed that the obtained cells positively expressed CD29 (99.98%), CD90 (98.75%), CD105 (99.99%), and CD44 (99.97%), which were markers of mesenchymal stem cells, while markers CD34 (0.06%) and CD45 (0.02%) for hematopoietic stem/progenitor cells were negative (Figure 2e). Vimentin and Nestin showed positive expression in immunofluorescence detection (Figure 2f).

**Figure 3:**
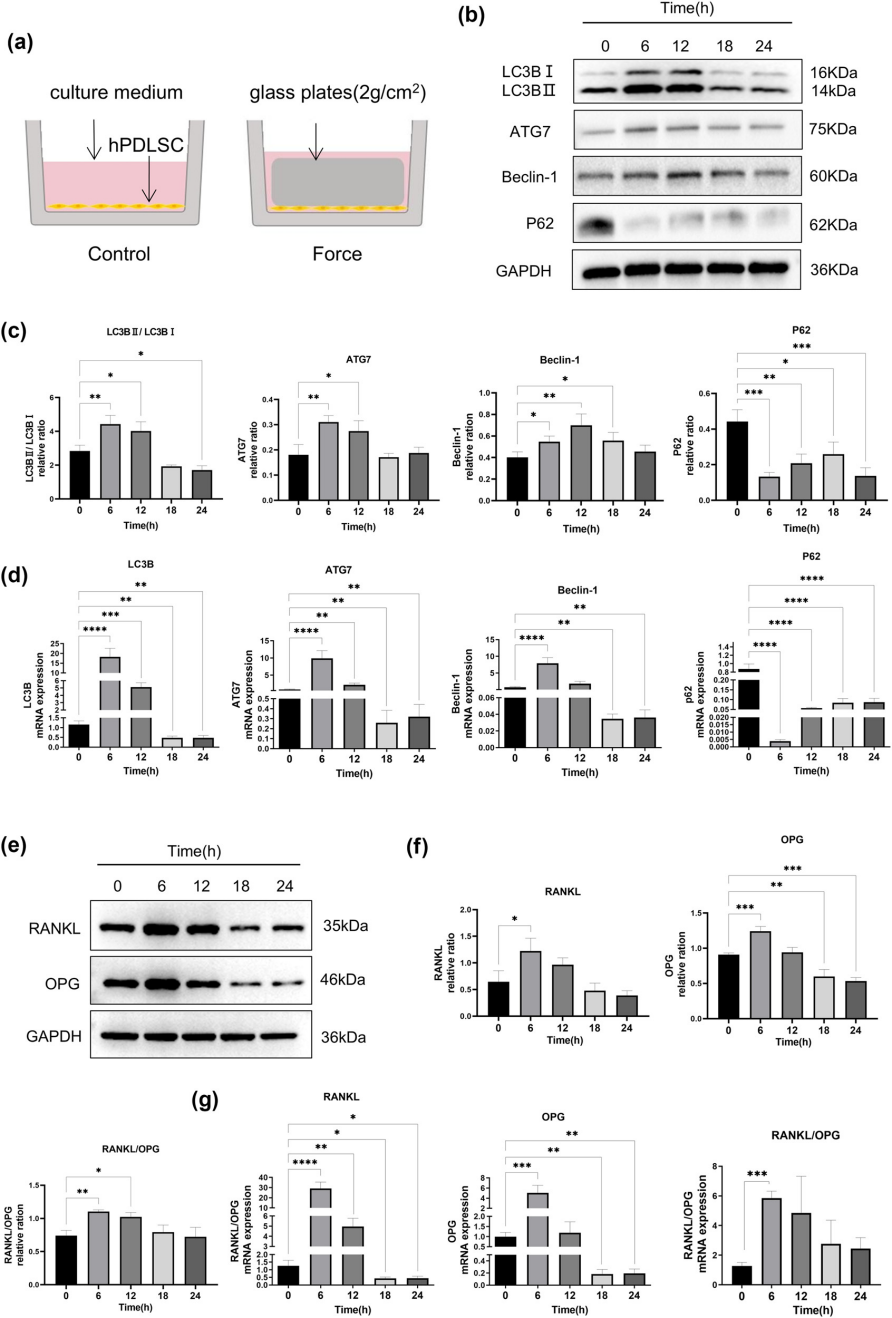
Effects of compressive stress on autophagy and RANKL/OPG expression in hPDLSCs. (a) Cell compressive stress model. (b)–(d) Western blot and RT-qPCR results of autophagy-related proteins, including LC3BⅡ/LC3BⅠ, ATG7, Beclin-1, and P62 after being cultured in the medium with a mechanical loading force of 2 g/cm^2^ for 0, 6, 12, 18, and 24 h. Autophagy-related proteins and mRNA were significantly activated at 6 and 12 h, and decreased at 18 and 24 h, with the first expression peak at 6 h. (e)–(g) Western blot and RT-qPCR results of RANKL and OPG expression after being cultured in the medium with a mechanical loading force of 2 g/cm^2^ for 0, 6, 12, 18, and 24 h. The RANKL and OPG expression significantly increased at 6 h, then decreased gradually, and the ratio of RANKL/OPG increased significantly at 6 h. *n* = 3; *p < 0.05; **p < 0.01; ***p < 0.001; ****p < 0.0001.

hPDLSCs were subjected to physiologic sustained mechanical load of 2 g/cm^2^ (Figure 3a). To evaluate the effects of the stress on the autophagy and RANKL/OPG expression of hPDLSCs, we detected the expression of autophagy-related factors, including LC3BⅡ/LC3BⅠ, ATG7, Beclin-1, and P62 by performing Western blotting (Figure 3b and c) and RT-qPCR (Figure 3d), as well as the expression of osteoclast-related factors RANKL and OPG (Figure 3e–g, e and f represent Western blot, while g represents RT-qPCR). The results showed that under stress, autophagy-related factors LC3BⅡ/LC3BⅠ, ATG7, and Beclin-1 were significantly upregulated, while P62 was significantly downregulated, and the overall autophagy flux of cells increased at 6 and 12 h. Under mechanical stress, the expression of RANKL and OPG demonstrated an initial increase followed by a decline. The RANKL/OPG ratio significantly increased at 6 h, indicating that under compression force, hPDLSCs tend to modulate osteoclast differentiation.

### 3.3 Effect of knockdown ATG7 gene on compressive stress-induced autophagy and RANKL/OPG expression in hPDLSCs

**Figure 4:**
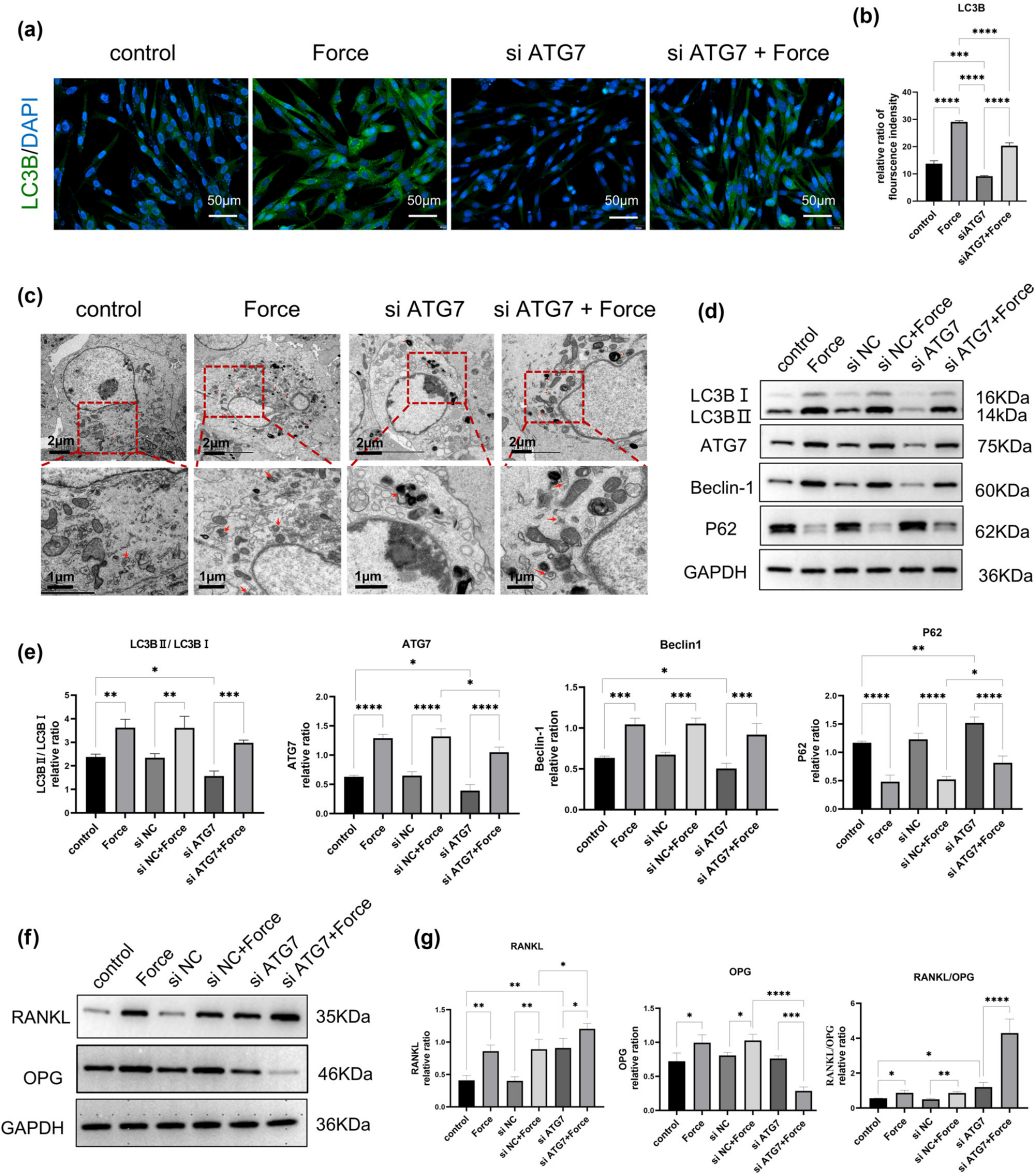
Knockdown of the ATG7 gene inhibits compressive stress-induced autophagy and promotes osteoclast differentiation in hPDLSCs. (a) and (b) The protein level of LC3B was detected by immunofluorescent staining. The immunofluorescence intensity of LC3B was enhanced under stress and decreased under siATG7 transfection. In hPDLSCs-siATG7 under the stress group, the immunofluorescence intensity enhanced again. (c) Autophagosomes were observed by TEM. The number of autophagosomes increased under stress, and decreased after ATG7 knockdown in hPDLSCs, which increased again by stress stimuli on hPDLSCs-siATG7. (d) and (e) Western blot results of autophagy-related genes and the quantitative analysis of protein expression levels. (f) and (g) Western blot results of RANKL/OPG expression and the quantitative analysis of protein expression levels. *n* = 3; *p < 0.05; **p < 0.01; ***p < 0.001; ****p < 0.0001.

To investigate the impact of ATG7 on hPDLSCs, we designed three pairs of primer sequences of siRNAs for screening the transfection efficiency of siATG7. Finally, we selected siRNA1, which showed the highest inhibition of ATG7 protein expression in the WB results for the subsequent experiment. The screening results are shown in Figure S1. We conducted immunofluorescence detection of LC3B expression. As shown in the results (Figure 4a and b), the protein expression of LC3B increased after 6 h of compressive stress, and decreased in the hPDLSDs-siATG7 group. When stress was applied to the hPDLSCs-siATG7 group, the intensity of immunofluorescence increased again. Transmission electron microscopy observation (Figure 4c) of autophagosomes in cells further confirmed that stress significantly increased the number of autophagosomes, while ATG7 knockdown significantly reduced the number of autophagosomes. After 6 h of continued stress, the number of autophagosomes in hPDLSCs-siATG7 increased again. The Western blotting (Figure 4d and e) results showed that after 6 h of compressive stress, autophagy-related factors in hPDLSCs were significantly upregulated, including LC3BⅡ/LC3BⅠ, ATG7, and Beclin-1. Notably, these markers showed an opposite trend in hPDLSCs-siATG7. In contrast, the expression level of P62 is downregulated in compressive stress and upregulated in hPDLSCs-siATG7. When stress was applied to the hPDLSCs-siATG7, the results indicate that compressive stress may partially reverse the effects of siATG7 transfection, characterized by a certain degree of upregulation of LC3BⅡ/LC3BⅠ, ATG7, and Beclin-1 and downregulation of P62.

Western blotting (Figure 4f and g) results showed that osteoclast-related factor RANKL and OPG exhibit different expression trends in the hPDLSCs-siATG7 group and hPDLSCs-siATG7 under the stress group. Specifically, compressive stress and ATG7 knockdown in hPDLSCs can both independently lead to an increase in RANKL expression. Furthermore, when stress loading was applied to hPDLSCs with ATG7 knockdown, a more significant increase in RANKL expression was observed. However, in the hPDLSCs-siATG7 group, there was no significant difference in the expression of OPG compared with the control group, but when the stress was applied to hPDLSCs-siATG7, the expression of OPG was significantly reduced. Notably, under compressive stress, hPDLSCs transfected with siATG7 exhibited a marked increase in the RANKL/OPG ratio.

### 3.4 Heterozygous ATG7^+/−^ rats enhanced OTM in the orthodontic model

**Figure 5:**
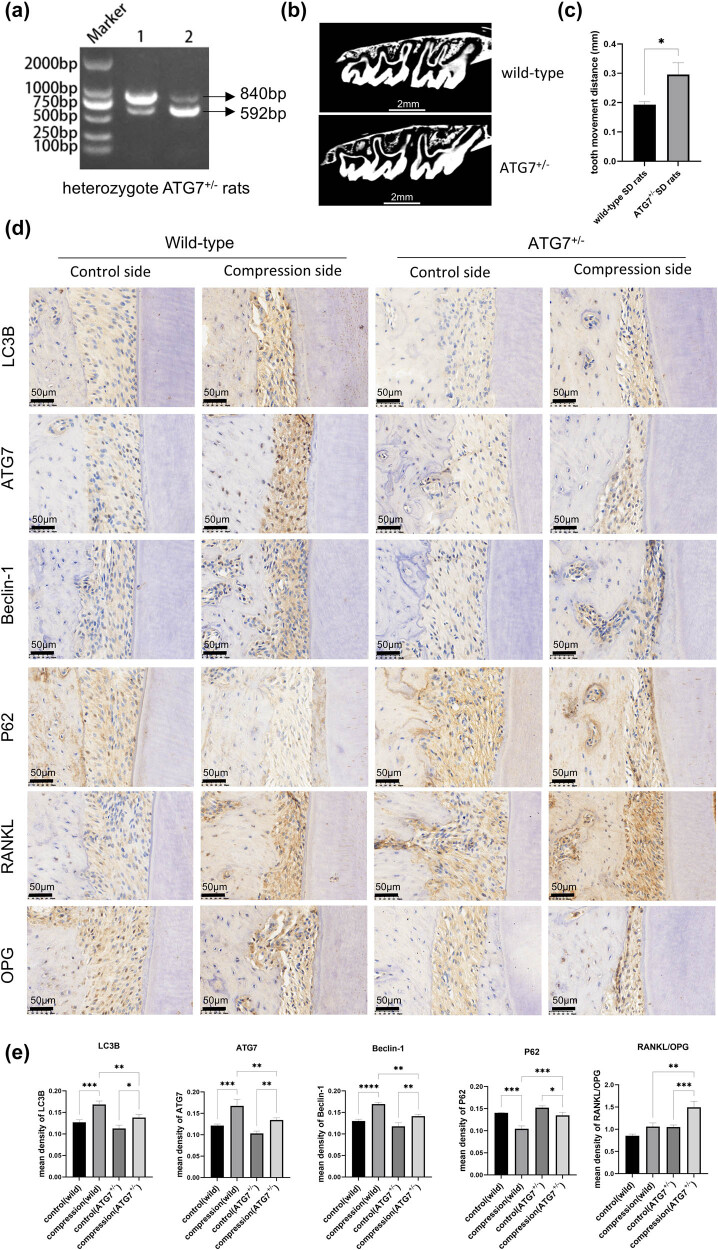
Heterozygous ATG7^+/−^ rats enhanced OTM in the orthodontic model. (a) Rats were identified by performing PCR. (b) and (c) Micro-CT scan and analysis. The results of tooth movement data are expressed as mean ± SD; the wild-type group was 0.193 ± 0.01 mm, and the ATG7^+/−^ group was 0.295 ± 0.04 mm. (d) and (e) Immunohistochemistry staining and its quantitative analysis for autophagy proteins, RANKL, and OPG. The autophagy-related proteins in the ATG7^+/−^ group were lower when compared to the wild-type group on the compression side, but with a significantly increased RANKL/OPG expression. *n* = 3 per group; samples were selected based on the technical quality for histological analysis. *p < 0.05; **p < 0.01; ***p < 0.001; ****p < 0.0001.

To elucidate the influence of the ATG7 gene on the OTM model, heterozygous rats with positive PCR results for both 840bp and 592bp bands were utilized in this study (Figure 5a). The Micro-CT measurement and analysis results indicated that the rate of OTM was considerably accelerated in the ATG7^+/−^ rats orthodontic model compared to that observed in wild-type rats (Figure 5b and c). The average tooth movement distance in wild-type SD rats was 0.193 mm, while the average distance in ATG7^+/−^ SD rats was 0.295 mm, with a significant difference observed between the two groups. We detected the expression levels of autophagy-related proteins in the ATG7^+/−^ rats group and wild-type rats group (Figure 5d and e). On the compression side, autophagy-related protein was elevated in both the wild-type group and the ATG7^+/−^ group, as evidenced by the upregulation of LC3B, ATG7, Beclin-1, and the downregulation of P62. However, the autophagy-related protein induced by compressive force in the ATG7^+/−^ group was significantly lower compared to the wild-type group. On the control side, although the autophagy-related protein of the ATG7^+/−^ group was lower than that of the wild-type group, there was no significant difference between the two groups. Immunohistochemical staining (Figure 5d and e) for RANKL and OPG demonstrated that the RANKL/OPG expression increased on the compression side both in the wild-type group and the ATG7^+/−^ group. In the ATG7^+/−^ group, both the control and stress sides exhibited an increase of RANKL/OPG ratio compared to the wild-type group. Notably, on the compressive stress side of the ATG7^+/−^ group, the expression of RANKL/OPG was significantly increased.

## 4 Discussion

**Figure 6:**
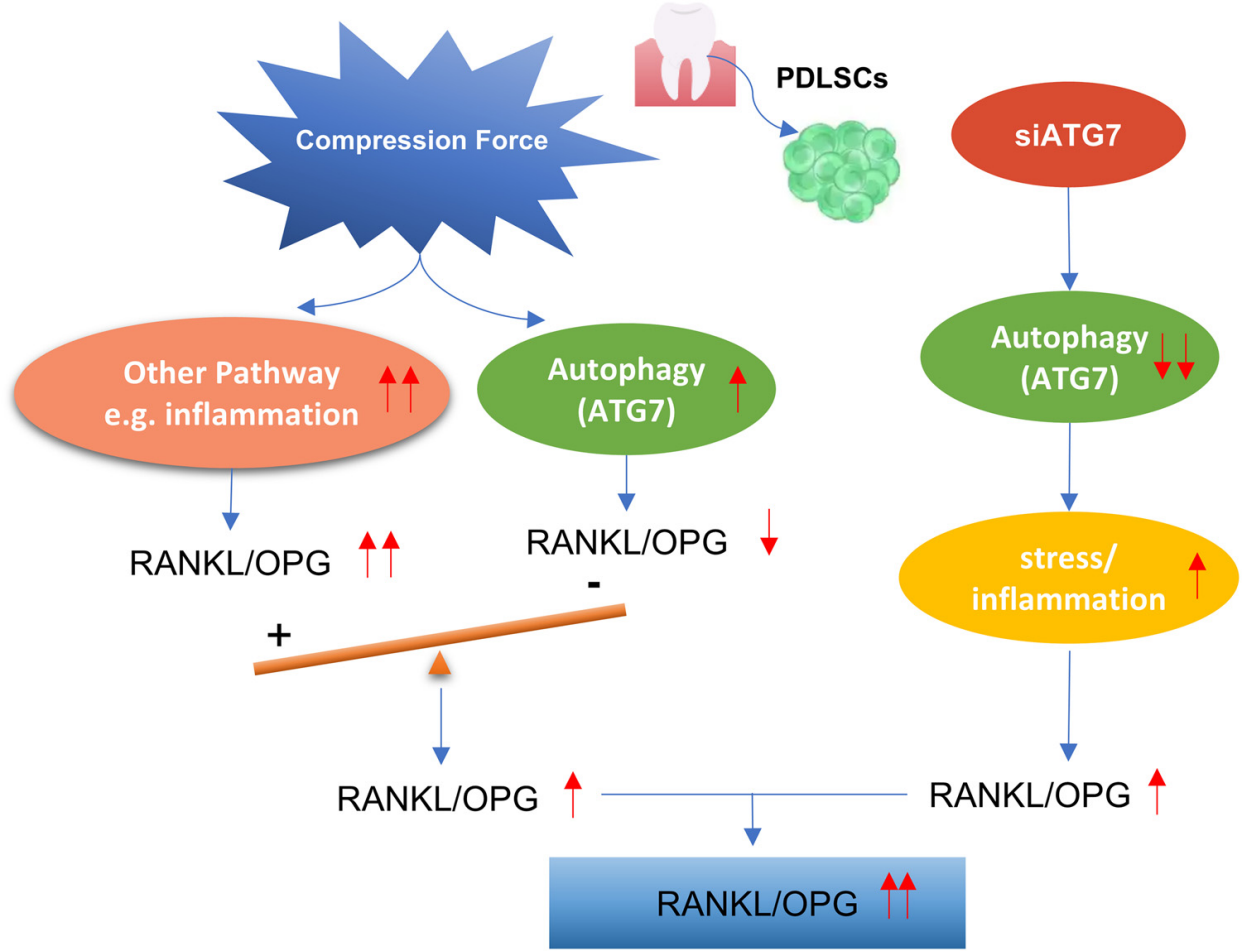
Schematic diagram of the relationship between the compression force, ATG7, and RANKL/OPG.

This study was designed to identify whether ATG7 modulated periodontium remodeling under compressive stress. In our experiments, mechanical compressive stress was found to upregulate autophagy-related proteins, including ATG7, and concurrently increased the RANKL/OPG ratio. Paradoxically, we also observed that knockdown of ATG7 led to a further elevation in the RANKL/OPG ratio, both *in vitro* and *in vivo*. We observed that these findings appear contradictory. However, mechanical compression is known to activate multiple cellular pathways in periodontal ligament cells, not limited to autophagy. Notably, it also triggers inflammatory and hypoxic responses [4,5,9], both of which are well-documented inducers of RANKL expression. While ATG7 and autophagy may negatively regulate osteoclastogenesis under certain conditions, this suppressive effect may be overridden by pro-inflammatory signaling cascades under mechanical load. In this context, the increase in ATG7 expression may exert a compensatory, protective role against stress but may not be sufficient to counteract the dominant RANKL-promoting effects of inflammation. As a result, despite the elevated expression of ATG7, the overall expression of RANKL/OPG under compressive stress remains increased. Conversely, suppression of ATG7 may impair autophagy and exacerbate cellular stress, potentially enhancing inflammation-driven RANKL expression through pathways such as NF-κB or MAPKs. Thus, the upregulation of RANKL/OPG observed in both ATG7 upregulation and downregulation contexts likely arises from the integration of multiple, competing signals. Consequently, when ATG7 expression is inhibited, the application of mechanical stress further amplifies RANKL/OPG expression, which may eventually affect the OTM speed (Figure 6). We acknowledge that the precise mechanistic link between ATG7 and RANKL/OPG regulation remains to be fully elucidated. In particular, overexpression studies of ATG7 in hPDLSCs may help clarify whether ATG7 directly suppresses RANKL expression. This represents an important direction for our future work.

PDLSCs play an essential role in the formation, regeneration, and remodeling of periodontal tissue. They are recognized as a critical stem cell population for periodontal tissue repair and regenerative therapy [24,25]. To explore the effects of mechanical loading on periodontal tissue, various forces have been applied to hPDLSCs, including fluid shear stress, centrifugal force, tensile stress, and compressive stress [26–28]. Periodontal ligament cells are considered the primary cellular receptors for orthodontic mechanical signals in periodontal tissues. Applying physiological orthodontic compressive stress to human periodontal ligament cells has been shown to induce the expression of genes associated with bone remodeling, inflammation, extracellular matrix remodeling, and angiogenesis [29]. Although PDLSCs are widely used as an *in vitro* model to investigate cellular responses during OTM, their application has inherent limitations. The periodontal microenvironment *in vivo* is highly complex and involves not only PDLSCs but also osteoblasts, osteoclasts, endothelial cells, immune cells, and mechanical forces from the surrounding tissue and vasculature. PDLSC monocultures lack these dynamic interactions, which may affect the translatability of the results. Thus, while PDLSCs provide useful mechanistic insights, further validation in animal models is necessary.

In our *in vitro* experimental results, applying a compressive force of 2 g/cm^2^ to hPDLSCs significantly modulated autophagy-related factors LC3BⅡ/LC3BⅠ, ATG7, and Beclin-1 upregulated, while P62 was downregulated. The observed differential expression patterns of autophagy-related markers under orthodontic force can be attributed to their distinct functional roles in the autophagic process. Beclin-1 and ATG7 are upstream regulators essential for autophagosome initiation and expansion, respectively. LC3B, particularly its lipidated form LC3BII, associates with autophagosome membranes and serves as a marker for autophagosome number [15]. In contrast, p62 acts as a cargo receptor that is degraded during autophagy; thus, its accumulation typically indicates impaired autophagic flux, while a decrease suggests active autophagy [30]. Consistent with our findings, other studies have also demonstrated that autophagy is a rapidly activated protective physiological response to adapt to compressive stress [31,32]. As for the ATG7 expression peaks at 6 h in our study, subsequent experiments were conducted with stress loading for 6 h.

RANKL is a key cytokine that binds to its receptor RANK on osteoclast precursors, directly stimulating their differentiation, activation, and survival. OPG is a soluble decoy receptor secreted by osteoblasts and stromal cells that binds RANKL and prevents it from interacting with RANK [33]. The RANKL/OPG does not directly measure osteoclast formation or activity, but its ratio reflects the potential environmental signaling. An increased RANKL/OPG ratio is indicative of enhanced osteoclastogenesis and bone resorption, which are fundamental processes that facilitate tooth movement. Therefore, we used changes in the RANKL/OPG ratio to reflect osteoclast differentiation activity in the periodontium and found that the rate of tooth movement after mechanical stress or autophagy deficiency is of great significance. In this study, we observed that compressive stress significantly increased the RANKL/OPG ratio in hPDLSCs, particularly under ATG7 knockdown conditions, suggesting that autophagy may negatively regulate pro-osteoclastic signaling. This finding is supported by previous research showing that compressive force upregulates RANKL in a time- and force-dependent manner in PDL cells, thereby enhancing osteoclastogenesis in co-culture systems with osteoclast precursors [34,35]. Our multi-modal analyses, including Western blotting, LC3B immunofluorescence, and transmission electron microscopy, confirmed that ATG7 knockdown significantly suppressed autophagy in hPDLSCs, while mechanical loading partially restored autophagic activity. This partial restoration may reflect a stress-induced compensatory mechanism that allows limited autophagic flux despite ATG7 suppression. Mechanistically, our results align with previous studies, indicating that silencing autophagy genes such as ATG5, ATG7, and Beclin-1 impairs mesenchymal stem cell osteogenesis and promotes osteoclastogenic environments [19,36]. *In vivo*, osteoblast-specific ATG7 cKO mice displayed reduced bone mass and elevated RANKL levels, confirming the dual role of ATG7 in both osteoblast formation and osteoclast inhibition [17]. Taken together, our findings and existing literature suggest that ATG7-mediated autophagy acts as a key modulator in periodontal tissue homeostasis and maintaining a balanced bone remodeling process under mechanical stress. A limitation of this study is the use of siRNA-mediated ATG7 knockdown, which may result in partial inhibition of autophagy due to suboptimal transfection efficiency. Future studies using CRISPR/Cas9-mediated ATG7 knockout models may provide more definitive insights into the mechanistic role of autophagy in hPDLSCs under mechanical stress. However, excessive activation or inhibition of autophagy can also impact bone homeostasis. It is clear that autophagy and bone homeostasis are interdependent and mutually regulated.

Orthodontic force application typically induces tooth movement through three distinct phases: the initial phase of rapid tooth movement immediately following force application (usually the first three days), the subsequent phase of slowed tooth movement, and the final phase of linear tooth movement [22,37]. In our experimental findings, the autophagic activity on the compression side exhibited a relative peak around day 3. This elevation in autophagy is considered indicative of active periodontal tissue remodeling, as autophagy plays a critical role in maintaining tissue homeostasis and responding to mechanical stress, which are essential processes during OTM [7,8]. Thus, investigating molecular expression changes in periodontal ligament tissues at this time point provides representative insight into the remodeling response. Based on these observations, we conducted our subsequent *in vivo* OTM experiment for 3 days. Although our study primarily focused on autophagy-related markers, other indicators of periodontal remodeling, such as ALP, RUNX2, RANKL, OPG, matrix metalloproteinase-9 (MMP-9), and TRAP, could be incorporated in future studies to provide a more comprehensive evaluation of the remodeling process.

ATG7 deficiency impairs the degradation of autophagosomal inner membranes following autophagosome-lysosome fusion [38], and this disruption of autophagy has been demonstrated in yeast, mice, and humans [39–41]. We used heterozygote rats in this study because the survival rate of homozygous rats was low. Although tissue-specific conditional knockout models are available in rats, constructing a periodontal ligament-specific cKO model remains technically challenging. Additionally, isolating sufficient PDL tissue from rats for downstream analyses is also difficult. While heterozygous rats showed no remarkable differences in physical appearance compared to wild-type rats at 7 weeks of age, their body weights were significantly lower (wild-type: 205.5 ± 9.88 g vs ATG7^+/-^: 171.5 ± 10.25 g), and relevant results are shown in Figure S2. The *in vivo* results shown in Figure 5d and e demonstrated that compared to wild-type SD rats, ATG7^+/−^ SD rats exhibited slightly lower levels of autophagy markers, including LC3B, Beclin-1, and ATG7, as well as a slight accumulation of P62 in the periodontal tissues. However, these differences were not statistically significant. However, on the compression force side, the ATG7^+/−^ SD rats showed a significant decline in the autophagy level, which indicated that the autophagy-related protein of heterozygote rats was affected after stress loading. Additionally, these rats showed an increased RANKL/OPG ratio on the compression side and a significantly accelerated rate of tooth movement. The absolute increase in tooth movement during the first 3 days was 0.102 mm; this degree of enhancement is comparable to that achieved by some non-invasive approaches currently under investigation or clinical use, such as low-level laser therapy or vibration devices [42,43]. A limitation of this study is the reduced number of analyzed samples (*n* = 3 per group), which was due to technical constraints. Specifically, during histological processing, tissue folding, sectioning artifacts, or staining inconsistencies led to the exclusion of several samples from quantitative analysis. However, a post hoc power analysis of the ooth movement result indicated that the observed effect size was sufficiently large (Cohen’s *d* = 3.49), yielding a statistical power of 0.996, thereby supporting the reliability of our findings. Nonetheless, we acknowledge that the high attrition rate may compromise the representativeness of the retained samples and could affect the reproducibility of the results. Future studies will aim to improve tissue preparation protocols, such as optimizing fixation, embedding orientation, and sectioning techniques, to reduce sample loss. Additionally, larger initial cohorts should be considered to ensure adequate sample availability after processing.

Nonetheless, we acknowledge that the minimal baseline differences in ATG7 expression between wild-type and ATG7^+/−^ rats may limit the interpretation of gene dosage effects. Future studies employing conditional knockout models or more precise quantitative measurements of ATG7 protein levels under stress conditions would provide stronger validation of the biological relevance of ATG7 in OTM.

Taken together, these results provide strong evidence that ATG7 plays a regulatory role in RANKL/OPG and OTM. However, it would be better if we had sufficient homozygous rats to investigate the dynamic variations of autophagy and RANKL/OPG under different loading times. We also recognize that the ATG7^+/−^ genotype itself is not directly translatable into a clinical intervention. To address this, we propose that our findings open up a potential avenue for targeted modulation of autophagy pathways as a therapeutic strategy. Pharmacologic or gene-based approaches that locally modulate ATG7 expression or autophagy level at the periodontal site could, in theory, replicate the effects observed in our model without systemic alteration of gene expression. Such approaches would need to be carefully controlled to avoid unwanted side effects, and future studies will be necessary to optimize delivery methods and evaluate long-term safety.

## 5 Conclusion

Knockdown of ATG7-modulated RANKL expression in periodontal ligament cells, under compressive force, was associated with a significant increase in the RANKL/OPG ratio both *in vivo* and *in vitro*. This was accompanied by a greater extent of OTM. Our findings suggest that ATG7-mediated autophagy may play a regulatory role in alveolar bone remodeling during orthodontic force application, providing a potential experimental basis for strategies aimed at accelerating tooth movement.

## Supplementary Material

Supplementary Figure
