# Supplementary material for: Inhibition of ATG7 promotes orthodontic tooth movement by regulating the RANKL/OPG ratio under compression force
Source: Open Med (Wars). 2025 Sep 12;20(1):20251252. doi: 10.1515/med-2025-1252 (PMC12452073; doi:10.1515/med-2025-1252)
Supplement: Supplementary Figure [file med-2025-1252-sm.pdf]

Supplementary material

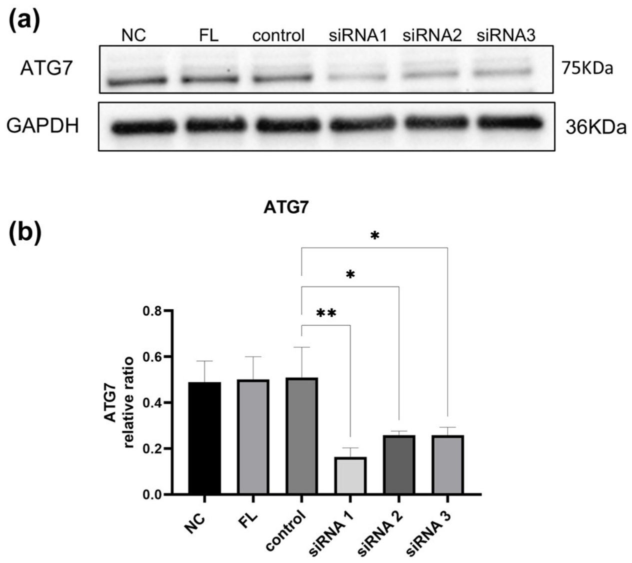

Figure S1: Screening and validation of ATG7-targeting siRNAs by Western blot. (a) Western blot result of ATG7 protein expression. The experiment included the following groups: negative control siRNA (NC), fluorescent-labeled siRNA control (FL), untreated cells (C), siRNA1-ATG7 (siRNA1), siRNA2-ATG7 (siRNA2), siRNA3-ATG7 (siRNA3). (b) The quantitative analysis of protein expression level. $n = 3$; $*p < 0.05$; $**p < 0.01$.

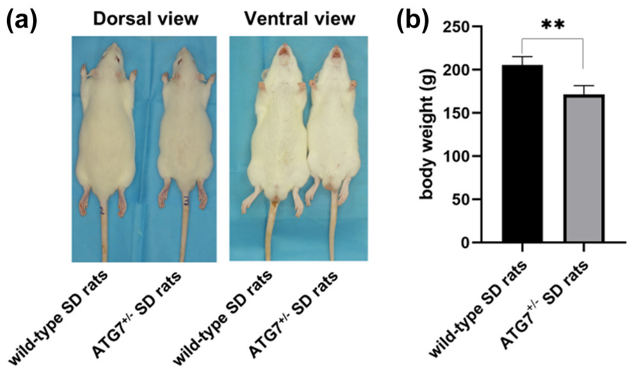

Figure S2: Comparison of body appearance and weight between wild-type and ATG7$^{+/-}$ SD rats. (a) Representative images showing the dorsal and ventral views of wild-type SD rats and ATG7$^{+/-}$ SD rats. (b) Body weight comparison between wild-type SD rats and ATG7$^{+/-}$ SD rats. The result were expressed as mean ± SD, the wild-type group was 205.5 ± 9.88 g, and the ATG7$^{+/-}$ group was 171.5 ± 10.25g. $n = 6$ per group, $**p < 0.01$.
